# Sample size in quantitative instrument validation studies: A systematic review of articles published in Scopus, 2021

**DOI:** 10.1016/j.heliyon.2022.e12223

**Published:** 2022-12-12

**Authors:** Michael White

**Affiliations:** Facultad de Ciencias Humanas y Educación, Universidad Peruana Unión, Km 19 Carretera Central, Ñaña, Lurigancho, Lima 15, Peru

**Keywords:** Sample size, Instrument validation, Systematic review

## Abstract

**Background:**

Due to the range of conflicting criteria regarding minimum sample size needed for a scale/questionnaire validation study, the objective of this review is to analyze sample sizes used in published journal articles to contribute a pragmatic perspective to the discussion on sample sizes.

**Methods:**

A sample of 1999 articles published in a Scopus-indexed journal about the validation of a scale or questionnaire during 2021 were analyzed for this study. Abstracts from these articles were tabulated by two data entry professionals and any discrepancies were reviewed by the author. The sample size data was grouped by highest quartile of the journal publishing the article and further sub-categorized based on the inclusion of medical patients or students in each study's population.

**Results:**

From the total sample, 1750 articles provided sufficient information in their summary to determine the sample size used. Of these, the majority were published in quartile 1 (784) and quartile 2 (620) journals. Mean values by quartile ranged from 389 (quartile 3) to 2032 (quartile 1), but extreme outliers limited the usefulness of the simple mean. Thus, outlier-removed means were calculated, and in most cases, these sample size values were higher for studies involving students and lower for studies involving patients.

**Discussion:**

This study is limited by its focus on a single database and by including all phases of validation from initial quantitative instrument design studies (which tend to have the lowest sample sizes) up to international macro-studies (which can have hundreds of thousands of participants.) Nevertheless, the results of this study provide an additional practical perspective for the academic discussion regarding minimum sample size based on accepted practice.

## 1 Introduction

### 1.1 Rationale

A wide range of fields use quantitative instruments such as scales and questionnaires to collect information for research studies, but there are different and conflicting opinions regarding how many people must participate in such a study. Over the years, there have been several proposals for “rules of thumb” such as 10 participants for every question in the instrument (Everitt, 1975), a simple flat minimum (with a few caveats like good model fit) of 100 participants (Kline, 1994), or a range from 50 as very poor through 300 as good up to 1000 or more as excellent (Comrey and Lee, 1992). Additionally, there are a variety of formulas that have been proposed to calculate sample size, with some going so far as to provide software or web applications to run these calculations, as is the case with the online calculator for structural equation modeling (SEM) studies made available by Soper (n.d.). These theoretical and mathematical approaches provide important context for the discussion regarding sample size, but they have not produced universally accepted answers. As part of this academic conversation, some studies are beginning to review the sample sizes used (and the justification given for them) in already published articles.

Some studies have reviewed the explanations given for sample sizes in articles from certain fields, as in the study carried out for Patient-Reported Outcome (PRO) measures by Anthoine et al. (2014), where they observed with concern how few studies (less than 10% of those included in their review) included an a priori determination of the sample size. In their study, mean sample size was shown to be quite susceptible to outliers, where they reported a mean of 509, SD of 1094, and median of 207. They even go so far as to mention how the results could lack sufficient power and precision due to their unfounded sample sizes. This effectively places them at odds with other articles in the conversation about sample sizes which reject the emphasis on power analysis and the need for a priori sample sizes, such as the article by Silva Ayçaguer and Alonso Galbán (2013). Both highlight the lack of a priori sample size determination among published articles, but Anthoine et al. (2014) suggest it is due to the lack of accepted and validated ways to calculate the sample size whereas Silva Ayçaguer and Alonso Galbán (2013) argue that such a determination of sample size is unnecessary and even irrational.

Anthoine et al. (2014) call for simulation studies to provide more information to help set a minimum sample size for different circumstances, and some such studies have indeed been done. Mundfrom et al. (2009) used simulation studies to evaluate the minimum sample size needed for factor analysis of a single instrument, and found that a higher number of factors leads to a higher sample size requirement, whereas a higher number of items per factor leads to a lower minimum sample size. Thus, a single factor scale with at least 9 items can have a sample as low as 50, but a six-factor short form instrument with only 3 items for each factor would need a sample size of 3800. Similarly, Wolf et al. (2013) evaluated minimum sample size requirements for SEM, and their results for confirmatory factor analysis (CFA) were similar to those found in the previous study for exploratory factor analysis (EFA), in that a higher number of factors and lower number of items per factor results in higher minimum sample sizes for CFA, but adding that as factor loadings approach 1, the minimum sample size decreases, with loadings of .80 requiring one third the sample size of .50 loadings.

While simulation studies are arguably more accurate than other previous attempts to set rules for sample sizes, the reality is that sample sizes are far from standardized and objective. Over ten years ago, Bacchetti (2010) reflected on the way small changes in sample size formulas can lead to drastically different minimum sample size numbers and concluded, “Whether a sample size justification passes peer review therefore depends on arbitrary reviewer discretion, which is a bad situation for a process meant to be fair.” This highlights a practical implication of this conversation regarding sample size within academic literature, namely that peer reviewers can hold to different views than the authors of a paper, and thus call into question the validity of the sample size used. Given the lack of consensus regarding minimum sample size, such an observation from a reviewer can be difficult for authors to answer.

Thus, there is a need for further discussion regarding minimum sample sizes, with additional perspectives. This study does not attempt to provide a theoretical framework for calculating sample size, nor does it use simulation studies to present more data regarding sample size needs in certain scenarios. For a theoretical perspective including p value, power, and effect, see Whitley and Ball (2002) and for a summary of a wide range of approaches including Monte Carlo simulation studies regarding sample size, see Kyriazos and Kyriazos (2018). Given the literature already available from both theoretical and simulation-based methods, this study focuses on examples of studies that have already been published in Scopus about quantitative instrument validation so as to provide information that can help researchers make an informed decision about the sample size they should aim for in their own studies. Although this information could also help inform related study designs, such as correlational and descriptive studies, it is most applicable to instrument validation studies. Based on the current of thought expressed by publications like Silva Ayçaguer and Alonso Galbán (2013) and Bacchetti (2010), this study makes no attempt to judge or categorize the analyzed articles into “sufficient” or “insufficient” sample sizes. Rather, all articles are taken as valid for their own purposes, given that they were published in indexed journals with quality standards in place, and the results are presented for analysis and guidance for future studies.

### 1.2 Objective

The objective of this study, then, is to review a large sample of journal articles that were published recently in a database with worldwide recognition to explore trends in the sample sizes used based on quartile of the journal where the article was published and the inclusion of students or patients in the study and thus arrive at some tentative suggestions for sample size for quantitative instrument validation studies that would coincide with other already-published articles. The database chosen for this project was Scopus and the year for articles to be included in the study was 2021.

## 2 Method

This is a systematic review of journal articles about the validation of an instrument, including both new instruments and translation or adaptation studies, published during 2021 in a journal indexed by Scopus.

Considering the difficulties and highly conflicting perspectives regarding sample size calculation, and even whether it should be calculated at all, this study follows the practical advice presented in studies like Bacchetti's (2010) and seeks to include a large enough sample so as to help account for outliers which can easily skew descriptive statistics such as the mean. It should be noted that the meta-irony of trying to decide how large of a sample is large enough to contribute to a discussion about how large a sample size should be was not lost on the author of this study. Instead, the database used for the analysis itself provided the sample size; Scopus, as of the time this study's advanced search was run, limits the export of results into a comma separated values (CSV) file at 2000 rows, meaning 1 row is used for the headers for each column and then 1999 search results can be included. Thus, this study analyzed the abstracts of 1999 publications in Scopus to find the sample size used in each study. Further analysis based on the information available from Scopus about the journals involved and other notable words found in the abstracts were used to add additional context to the results.

### 2.1 Eligibility criteria

Studies regarding factor analysis or psychometric analysis for new or newly translated scales, questionnaires, tests, or inventories in order to show their reliability and/or validity were included in the study. Systematic reviews were excluded from the study. Only articles published in journals were included, thus excluding conference proceedings as well as articles in press.

### 2.2 Information sources

An advanced search was conducted on December 8, 2021 in Scopus to include instrument validation articles which had been published in 2021.

### 2.3 Search strategy

The exact search string used is shown below:

TITLE-ABS-KEY ("factor analysis" OR "factorial analysis" OR psychometric) AND TITLE-ABS-KEY (new OR design OR translat∗) AND TITLE-ABS-KEY (scale OR questionnaire OR quantitative OR test OR inventory) AND TITLE-ABS-KEY (α OR alpha OR reliab∗ OR valid∗) AND NOT TITLE-ABS-KEY (systemat∗) AND (LIMIT-TO (PUBYEAR, 2021) OR LIMIT-TO (PUBYEAR, all)) AND (LIMIT-TO (SRCTYPE, "j")) AND (LIMIT-TO (DOCTYPE, "ar")) AND (LIMIT-TO (PUBSTAGE, "final"))

This search returned 2530 document results, and due to the aforementioned limitations of the Scopus platform, only 1999 were able to be exported into a CSV file.

### 2.4 Data preparation

The comma separated value (CSV) file from the Scopus advanced search was opened in Excel and extra data from the Scopus master journal list (available for download from the Sources search area when the user is logged in with a Scopus license) was added by linking the column with the journal's name from the exported articles with the master journal list. A column for highest quartile for the journal and another for whether or not the journal is in the Scopus Top 10% list were added using this extra information. Two additional columns were added to identify articles which included students or patients in their samples. The code in Excel (run against the column with the article abstract) is as follows, first for patients and then for students:

= OR(ISNUMBER(((SEARCH("patient",S2)))),ISNUMBER(SEARCH("diagnos",S2)))

= OR(ISNUMBER(((SEARCH("student",S2)))),ISNUMBER(SEARCH("undergrad",S2)))

These Excel formulas search for either of two common terms (patient or diagnosis for the patient group, and student or undergraduate for the student group) to identify article abstracts with these groups in their samples.

### 2.5 Selection and data collection process

The 1999 journal article abstracts were reviewed by two different data input professionals, each with over 3 years of experience and over 75 successful freelancing jobs completed. Both were hired using the *fiverr* platform for freelancers. Two columns were added to the Excel file with the sample size identified by each reviewer, and a third column was used to check if the two sample sizes coincided. A total of 433 discrepancies between the two reviewers were identified, and the author personally reviewed each discrepant abstract to identify the correct sample size.

### 2.6 Data items

For studies with more than one sample size given as part of the abstract, the following criteria were used. First, in the case of separate samples used for different levels of the analysis, such as Exploratory Factor Analysis (EFA) and Confirmatory Factor Analysis (CFA), only the highest sample size was included to avoid bias compared to other studies that only report one level of analysis. Second, for abstracts which broke down a large sample into subsamples (for example, number of diagnosed patients in one group and number of general public in another group), the total sample was the sum of the various subsamples so as to avoid bias compared to studies which did not provide detailed information about their sample by category in their abstract.

### 2.7 Study selection

Table 1 provides a summary of articles by journal quartile, including how many articles in each quartile used patients or students in their study and how many provided information in their abstract about the sample size used.

**Table 1:** Summary of articles included in the study.

Quartile	1		2		3		4		#N/A	
	Yes	No	Yes	No	Yes	No	Yes	No	Yes	No
Patients	295	594	231	467	83	123	22	65	46	73
Students	141	748	125	573	38	168	20	67	25	94
Sample Given	784	105	620	78	173	33	74	13	99	20
Overall	889		698		206		87		119	

### 2.8 Effect measures and synthesis methods

Once the sample sizes were identified (or found to be lacking) for all 1999 journal articles, overall descriptive statistics were calculated (see Table 2). The articles were then split into new Excel sheets based on the top quartile of the journal which published the article. This enabled a more detailed analysis based on quartile, including mean, median, and mean with outliers excluded (using the Excel function TRIMMEAN). These results were also further subcategorized based on whether the study used patients or students in their sample. A Kruskal-Wallis test was performed with sample size as the dependent variable and quartile of the journal where the results were published as the factor, which resulted in an H value of 36.272 with 4 degrees of freedom and a p value < .001.

**Table 2:** Descriptive statistics of sample size by quartile.

	Consolidated Highest Sample Size				
	#N/A	1	2	3	4
Valid	99	784	620	173	74
Missing	20	105	78	33	13
Mode	200.000	200.000	200.000	200.000	400.000
Median	269.000	363.500	312.500	232.000	337.000
Mean	542.182	2032.166	695.835	389.000	413.838
Std. Deviation	1147.578	19213.853	3079.213	566.209	366.139
Skewness	6.176	18.643	21.234	5.068	1.738
Std. Error of Skewness	0.243	0.087	0.098	0.185	0.279
Kurtosis	43.588	387.965	494.996	34.668	2.923
Std. Error of Kurtosis	0.481	0.174	0.196	0.367	0.552
Shapiro-Wilk	0.354	0.062	0.112	0.525	0.812
P-value of Shapiro-Wilk	<.001	<.001	<.001	<.001	<.001
Minimum	25.000	12.000	15.000	10.000	20.000
Maximum	9608.000	441398.000	73056.000	5227.000	1624.000
25th percentile	157.500	200.750	179.500	122.000	157.750
50th percentile	269.000	363.500	312.500	232.000	337.000
75th percentile	450.000	708.000	590.750	416.000	461.250

**Figure 1:**
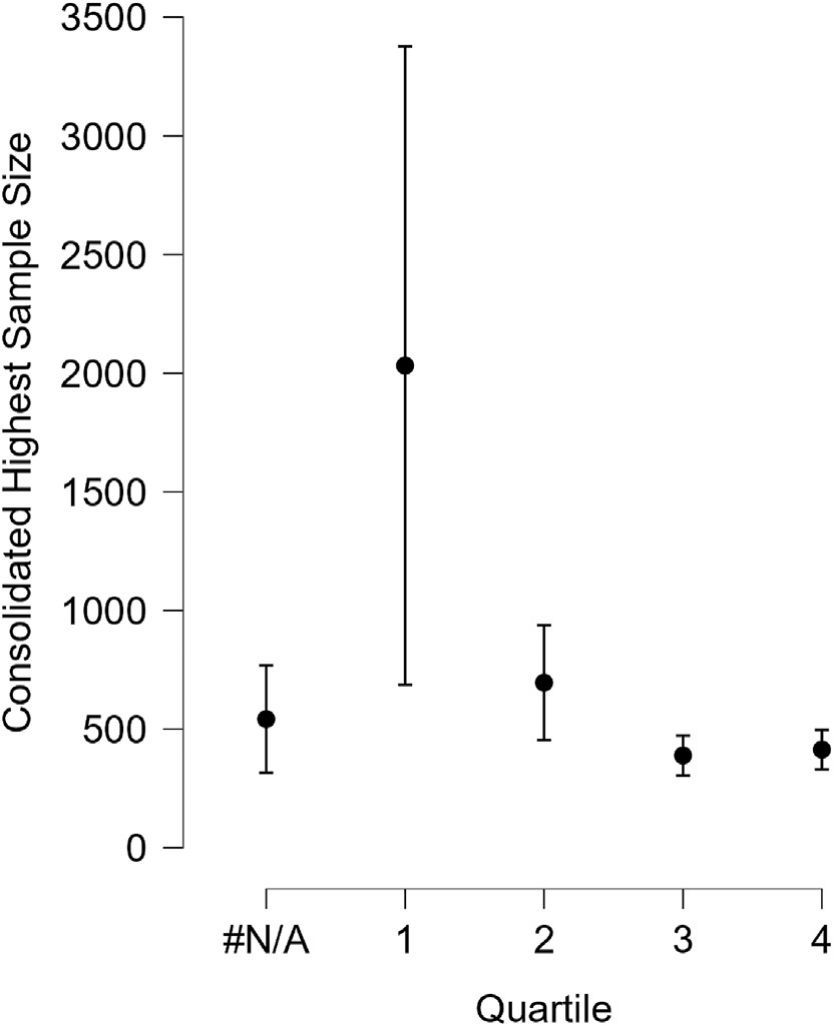
Interval plot of sample size by quartile.

Table 2 shows the descriptive statistics for the consolidated highest sample size, grouped by quartile of the journal where the article was published. The high standard deviations and large range between minimum and maximum values for each quartile should lead the averages to be interpreted with extreme caution. This can be further visualized in Figure 1.

Qualitative instrument design studies, where interviews or other qualitative techniques were used to create an initial bank of questions for a quantitative instrument, tend to make up the majority of the very small sample size studies. On the other extreme, studies that analyzed a piece of a large, even nation-wide or multi-national, study accounted for the majority of the largest sample sizes, with some in the hundreds of thousands of participants. With these outliers in mind, the Excel function TRIMMEAN was used to remove the most extreme 10% and 20% of sample sizes by category to provide a more accurate reflection of the most used sample sizes (Table 3 and Table 4, respectively.)

**Table 3:** Sample size with trim mean 10%.

Quartile	1		2		3		4		#N/A	
	Yes	No	Yes	No	Yes	No	Yes	No	Yes	No
Patients	409.55	572.67	290.63	512.12	197.46	394.36	199.00	462.67	209.05	546.75
Students	579.88	501.77	614.62	390.18	458.62	271.71	472.68	360.51	403.50	351.38
Overall	512.63		427.19		306.24		379.28		364.47

**Table 4:** Sample size with trim mean 20%.

Quartile	1		2		3		4		#N/A	
	Yes	No	Yes	No	Yes	No	Yes	No	Yes	No
Patients	356.99	518.60	262.02	467.46	189.10	367.95	194.65	431.62	204.80	467.41
Students	535.06	442.99	552.35	353.84	436.20	251.66	455.29	319.33	370.70	323.41
Overall	458.85		388.77		279.98		349.75		329.90	

## 3 Discussion

From a total of 1750 journal articles which included their sample size in their abstract, this study can provide some data which can inform decisions about sample sizes for instrument validation studies. A similar study which focused on 114 articles about the validation of health related quality of life scales found, “About 90% of the articles had a sample size ≥100, whereas 7% had a sample size ≥1000” (Anthoine et al., 2014). These results are similar to those found in this study. To add additional detail and provide a point of comparison, of the 1750 articles with sample sizes included in this study, 89% of the sample sizes were over 100, 53.5% were over 300, 31% were over 500, 19.8% were over 750, 13.9% were over 1000, and 4.6% were over 2000.

A few of the most noteworthy findings of this study are that higher quartiles (closer to 1) tend to have higher sample sizes, the participation of patients in a study tends to lower the sample size, whereas applying a study with students as the population tends to increase the sample size. Additionally, the extreme outliers make simple averages less useful, but averages with outliers excluded provide data to enable more informed decisions about commonly accepted sample sizes.

Regarding quartiles of the journals where the articles were published, one noteworthy finding is that the vast majority are in the top two quartiles. In other words, almost 80% of the articles included in this study were in the two highest categories of journals within the Scopus database. This suggests that researchers who conduct instrument validation studies often feel that their studies are “good enough” to get into the best journals. There is also a general tendency towards higher sample sizes in better quartile journals, with a notable anomaly wherein quartile 3 journals had an outlier-excluded average which was lower than both quartile 4 and those journals which do not yet have a quartile. In general, these results suggest that researchers who reached a lower sample size tended to send their articles to lower (closer to 4) quartile journals. The Kruskal-Wallis test showed a significant effect of journal quartile on sample size (H= 33.82, p < .001) which provides further evidence for a difference in sample size between articles published in higher compared to lower quartile journals.

One notable factor that needs to be addressed in the conversation about sample sizes is the ease of access and general quantity of the target population. This study highlights those articles which include students or patients as part of their sample. Given that many studies are conducted by researchers at educational facilities, who thus have ready access to a population of students, it is not surprising that studies involving student participants tend to have a higher sample size. Students are relatively easy to access and numerous, and thus there is a perceived need for a higher sample size. On the other hand, patients are generally less numerous, especially for rare conditions, so a whole hospital might only have a small number of eligible patients, and not all of them will necessarily participate in a study. Thus, it is not surprising that articles with patients in their sample tended to have lower sample sizes.

Finally, the question remains, what is the minimum sample size for a quantitative instrument validation study? First, the results of this study agree with a key lesson from Wolf et al. (2013) which used Monte Carlo simulation studies for Confirmatory Factor Analysis (CFA) and other Structural Equation Modeling (SEM) sample sizes, namely that the minimum sample size is best thought of as a range and it must be custom tailored to the study at hand. Second, the use of “rules of thumb” like 10 or 20 participants per item in the instrument, or absolute numbers like 100 or 1000, have been widely questioned for at least 15 years and, as Mundfrom et al. (2009) noted, it is not feasible to set a single, one-size-fits-all number for minimum sample size, especially given the effect that the number of factors and number of items has on the sample size requirement. Third, several studies, including the work of Bacchetti (2010) over a decade ago, highlight the frequent practical need to modify sample size calculations as presented in manuscripts for publication in order to reach the actual sample size used, leading to a level of academic dishonesty encouraged by an apparent taboo regarding honestly explaining the limitations that led to the sample size used. The current study agrees with this line of reasoning, and adds that a sample size based on what other studies have actually used is more transparent and direct than the use of formulas which are in turn based on somewhat subjective variables drawn from prior studies. In fact, this study is largely based on an idea expressed by Bacchetti (2010), who concluded, “A simple way to choose a sample size is to use one that has worked well in the past for similar or analogous studies.” Given the prevalence of outliers, a handful of studies is not enough to give an idea of what has “worked well in the past”, thus leading to the present study which analyzed 1750 articles.

With these caveats and comments in mind, a tentative suggestion for a sample size range would have to depend on the type of people who will participate in the study. If the study includes patients, a smaller sample size of approximately 250–350 would coincide with the findings of this study, whereas a study which includes students would need a larger sample of approximately 500–600. For studies among a general population (for example, adults from a certain country or city), an overall sample size of around 375–500 would be in keeping with the general trend for articles published in 2021.

To avoid confusion and misuse of these numbers, a few additional remarks are in order. First, this is for instrument validation studies, which tend to include exploratory factor analysis (EFA) and/or confirmatory factor analysis (CFA) or similar analyses, for a single instrument, and not for correlational studies. Although the similar nature of the studies might justify using similar sample sizes, additional research is needed to verify sample sizes in correlational studies. Second, these suggested sample size values are based on outlier-removed averages from articles published in quartile 1 and 2 journals, given that most instrument validation articles are published in these higher quartiles. Third, if a single study includes both EFA and CFA, these sample size numbers are for each stage of the analysis, and thus around twice the proposed numbers would be needed for a combination EFA and CFA study. Fourth, these ranges are most applicable for instruments with characteristics that have already been shown to require smaller sample sizes for validation studies. To use an extreme example, an instrument with a total of 30 items broken down into 3 items per factor over 10 factors would likely need a higher sample size than the ranges proposed by this study. Finally, a case can be made to go as low as 300 for a general sample size, since that would include over 50% of the articles reviewed in this study, but this ignores the different sample sizes used based on ease of access to the participants, and thus the lower values apply best to studies with groups such as patients with a certain diagnosis who are thus less numerous and/or more difficult to access than a general population.

This study has several limitations which should be mentioned. First, the types of analyses used in each article was not considered. A review of a few of the most extreme sample sizes suggests that most outliers are either qualitative studies used to design a quantitative instrument on the low end or subsections of larger studies at the high end. Future studies could use categories of analysis such as initial focus group, EFA, CFA, and larger multi-instrument studies to provide more specific information about common practice for sample sizes based on type of analysis used. Second, even among the majority of the articles which used EFA and/or CFA, no data was analyzed regarding the use of parametric or nonparametric tests and the influence that could have on sample size. Kalkan and Kelecioğlu (2016) compared the use of these different types of tests, and found that the sample size stabilized at different points for different types of analyses, thus future studies should take the type of tests used into account. Third, this study included only one database. Future studies should compare the sample sizes used in articles published in internationally acclaimed databases like Scopus and Web of Science with those published in regional or discipline-specific databases like SciELO, Latindex, ERIC, or JSTOR. Fourth, the number of items in the instruments used in the articles under review was not taken into account. There seems to be a trend towards shorter instruments with fewer factors, which helps explain the lower sample sizes compared to simulation studies like those of Mundfrom et al. (2009) which suggest high sample sizes needed for instruments with a large number of factors. Future studies should include data about the number of factors and the number of items in the instrument being validated. Finally, the articles published during the year included in this study, 2021, could have been affected by the COVID-19 pandemic. Future studies should evaluate sample sizes before and after the pandemic to see if it had any impact on outlier-removed average sample size or other related indicators.

While acknowledging these limitations, this study provides some additional information that can be useful for future studies and their decisions about sample size. Specifically, this study furthers the suggestion by Bacchetti (2010) to use the sample size of other similar articles as a basis for the sample size in a new proposed study. This was done by analyzing the sample sizes used in a total of 1750 articles related to quantitative instrument validation. Thus, the results of this study can provide tentative ranges of sample sizes that could be used for future instrument validation studies, based on the type of participants in the study, where sample sizes are generally lower with patients and higher with students as the population of study. No one study can provide a definitive answer to the sample size question, but the discussion should continue, and this study provides another perspective to add to the conversation.

## Declarations

### Author contribution statement

All authors listed have significantly contributed to the development and the writing of this article.

### Funding statement

This work was supported by Universidad Peruana Unión.

### Data availability statement

The authors do not have permission to share data.

### Declaration of interests statement

The authors declare no conflict of interest.

### Additional information

No additional information is available for this paper.
